# Leukocyte Ig-Like Receptors – A Model for MHC Class I Disease Associations

**DOI:** 10.3389/fimmu.2016.00281

**Published:** 2016-07-25

**Authors:** Laura Emily Hudson, Rachel Louise Allen

**Affiliations:** ^1^Institute for Infection and Immunity, St George’s, University of London, London, UK

**Keywords:** MHC, HLA, LILR, KIR, HIV

## Abstract

MHC class I (MHC-I) polymorphisms are associated with the outcome of some viral infections and autoimmune diseases. MHC-I proteins present antigenic peptides and are recognized by receptors on natural killer cells and cytotoxic T lymphocytes, thus enabling the immune system to detect self-antigens and eliminate targets lacking self or expressing foreign antigens. Recognition of MHC-I, however, extends beyond receptors on cytotoxic leukocytes. Members of the leukocyte Ig-like receptor (LILR) family are expressed on monocytic cells and can recognize both classical and non-classical MHC-I alleles. Despite their relatively broad specificity when compared to the T cell receptor or killer Ig-like receptors, variations in the strength of LILR binding between different MHC-I alleles have recently been shown to correlate with control of HIV infection. We suggest that LILR recognition may mediate MHC-I disease association in a manner that does not depend on a binary discrimination of self/non-self by cytotoxic cells. Instead, the effects of LILR activity following engagement by MHC-I may represent a “degrees of self” model, whereby strength of binding to different alleles determines the degree of influence exerted by these receptors on immune cell functions. LILRs are expressed by myelomonocytic cells and lymphocytes, extending their influence across antigen-presenting cell subsets including dendritic cells, macrophages, and B cells. They have been identified as important players in the response to infection, inflammatory diseases, and cancer, with recent literature to indicate that MHC-I recognition by these receptors and consequent allelic effects could extend an influence beyond the immune system.

## Introduction

MHC class I (MHC-I) proteins are characterized by a high level of polymorphism, with thousands of allelelic variants identified to date (1). Such extensive variation indicates powerful selection pressure to maintain a wide range of alleles. Disease associations for individual MHC-I alleles are well-documented. The most striking is that of HLA-B27, which is present in >90% of the patients with ankylosing spondylitis (2). MHC-I polymorphisms have also been shown to be associated with the outcome of viral infections, including the control of HIV infection (3), clearance of HCV infection (4, 5), and protection from dengue hemorrhagic fever following secondary infection with this virus (6).

Proposed mechanisms to explain classical MHC-I disease associations have focused on the functional role(s) of these proteins. The best characterized of these roles is MHC presentation of short antigenic peptides for recognition by the T cell receptor (TCR) on cytotoxic T cells (CTL). Thus, many studies have examined the nature of the peptides presented by disease-associated alleles and of T cell responses restricted by these alleles (7, 8). For example, a number of studies have examined the peptide specificities of HLA-B27 subtypes (9). In the context of HIV infection, a dominant HLA-B27 restricted viral peptide is thought to play a key role in the association of this allele with control of infection. Immune escape from the response against the dominant peptide results in a decrease in HIV-1 replication (10).

In humans, classical MHC-I are also recognized by members of the killer Ig-like receptor (KIR) family, which are encoded in the leukocyte receptor complex (LRC) on chromosome 19. KIR demonstrate allele (and in some cases, peptide) specificity (11), albeit at a lower level of precision for individual peptide/MHC complexes than that shown by classical TCR. KIR are expressed on natural killer (NK) cells and T cells where they inhibit the ability of these cytotoxic cells to lyse target cells that express self MHC-I alleles. As knowledge regarding their biology and MHC specificities has grown, KIR has been studied alongside MHC-I in conditions such as spondyloarthropathy, HIV, and HCV infections (5, 12, 13). There is considerable variation in KIR haplotypes such that any individual may not carry the relevant MHC ligand for every KIR receptor that they express and *vice versa*. A number of studies suggest that particular combinations of KIR and HLA alleles, believed to result in functional receptor/ligand interactions, are associated with protection from progression to AIDS following HIV infection (14).

A lesser-studied family of proteins encoded within the LRC is also capable of recognizing MHC class I. These leukocyte Ig-like receptors (LILR) do not appear to be involved in the cytolytic removal of targets bearing non-self MHC-I protein complexes (15). Instead, they are predominantly expressed on cells of the myelomonocytic lineage, and some of them show a broad specificity encompassing both classical and non-classical MHC-I (16). The observation that LILR vary in the strength of their binding to individual MHC-I alleles, however, raised the possibility that these innate immune receptors may contribute in some manner toward MHC-I disease associations (17). In support of this theory, a recent study of a large cohort of HIV-1 infected patients demonstrated that the overall binding strength of LILRB2 for the MHC-I haplotypes expressed by these individuals was positively associated with the level of viremia (18).

## Leukocyte Ig-Like Receptors

The various members of the LILR family are broadly categorized as inhibitory (LILRB) or activating (LILRA), according to the presence or absence of tyrosine-based signaling motifs in their cytoplasmic tail. In some cases, putative activating receptors have been shown to elicit inhibitory effects and *vice versa* for inhibitory receptors (19). Receptor engagement results in intracellular phosphorylation of the tyrosine-based motifs within the receptors themselves (LILRB) or on associated adaptor molecules (LILRA) (19). Downstream signaling events can be mediated by phosphatases such as SHP-1, SHP-2, and SHIP (20, 21) and vary according to the receptor and/or cellular context. For example, SHP-2 may mediate production of IL-6 *via* the NF-kB pathway following LILRB2 engagement on dendritic cells (22) or inhibition of the mTOR pathway following LILRB1 engagement on T lymphocytes (23).

There are multiple similarities between KIR and LILR in terms of Ig domain-based structure, gene location within the LRC, and ability to recognize MHC-I (15). Unlike their NK receptor counterparts, however, LILR orthologs (known as PIR) are found in rodents, where they demonstrate similar ligand binding, expression, and functional profiles (24, 25). This may indicate a higher degree of evolutionary conservation for LILR than for KIR, with bovine orthologs also identified (26) and similar proteins documented in chickens and fish (27, 28). Within the murine system, there is a single inhibitory receptor, PIR-B, and multiple activating receptors (PIR-A). PIRs are involved in the regulation of lymphocyte, antigen-presenting cell, and granulocyte functions (29), and their study has enabled the identification of functions for both these receptors and their human counterparts, such as the regulation of synaptic plasticity (30) and platelet activation by PIR-B and LILRB2 (31).

**Figure 1:**
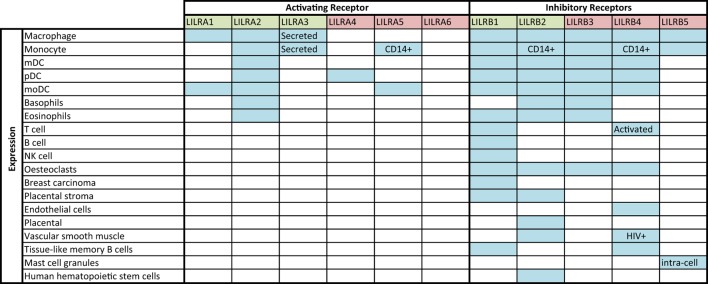
**LILR expression profile, according to literature**. Blue shaded squares indicate expression according to the literature (32–40); annotation within boxes indicates expression specifics (for example, observed during HIV infection or for a particular cell phenotype). Green denotes Group 1 LILR and red, Group 2 LILR.

Figure 1 shows the known expression profiles of LILR on leukocyte subsets according to current literature. The known expression profiles for LILR are not exhaustive; expression of individual members of the family has been documented for macrophages, B-cells, NK cells, and other non-immune cells (32–40). These receptors are, therefore, likely to have far-reaching effects on a range of immunological functions. Immune cells, which have yet to be characterized in full for LILR expression, include invariant NK (iNKT), gamma delta (γδ), regulatory (T_reg_) and T helper 17 (T_h_17) T-cells, B-cell subsets, as well as the various APC subsets and granulocytes.

Leukocyte Ig-like receptor activity can result in the upregulation or downregulation of both innate and adaptive functions with a range of effects on different cell types. For example, LILR and PIR have been shown to inhibit TLR-mediated functions of antigen-presenting cells such as inflammatory cytokine secretion (38, 41–43). Inhibitory LILR have been shown to inhibit the upregulation of co-stimulatory proteins on antigen-presenting cells (36, 44–46), thus favoring regulatory T cell responses (47–50). On lymphocytes, inhibitory LILR have been shown to inhibit T and B cell receptor signaling and downregulate antibody and cytokine production (51–53). Activating LILR have been shown to mediate monocyte activation and secretion of inflammatory cytokines (54) and on basophils to trigger release of histamine (55).

## MHC Recognition by LILR

Following the initial identification of LILRB1 as a receptor for self and viral MHC-I (56), structural studies predicted that several other members of the family would also recognize MHC-I (57). Members of the family were allocated into two groups on this basis, with Group 1 containing receptors predicted to bind MHC-I and Group 2 containing receptors that were not predicted to bind MHC-I (57). It was confirmed subsequently that the Group 1 members LILRA1, LILRA2, LILRA3, LILRB1, and LILRB2 can engage MHC-I (17, 58). Members of the LILR family vary in their MHC-I binding preferences. LILRB2 demonstrates the broadest specificity, with the ability to recognize all classical and non-classical self MHC-I alleles and forms tested to date. Although LILRB2 binds to both the α3 and β2m regions of the MHC-I antigen-presenting structure, the major portion of its binding site lies within the highly conserved α3 domain (59). The degree of interaction between this receptor and the α3 domain is sufficient to allow LILRB2 to bind open conformers of MHC-I, which lack β2m. In contrast, the major LILRB1 binding site lies within β2m, thus this receptor can only associate with β2m-associated MHC-I. Recognition of open MHC-I conformers has also been observed for LILRA1 and LILRA3, which were shown in one study to have stronger binding to open confomers than to β2m-associated MHC-I (17). These findings indicate that alternatively folded forms of MHC-I may play a functional role in the immune response. It is also important to note that the members of the LILR family may interact *in cis* with MHC-I on the cell surface, as been demonstrated for PIR-B and LILRB1 (60, 61).

Despite their broad specificity, LILRB1 and LILRB2 show variation in their strength of binding to different MHC-I alleles (17). Binding occurs predominantly through the D1-D2 domains of the receptor (57), but it has been suggested that secondary binding sites in the D3 and D4 domains may contribute to allelic variations in the strength of LILR binding (62). The potential importance of such variations was first highlighted by the observation that MHC-I complexes differing by only one amino acid in the bound peptide showed different affinities for LILRB2, which corresponded with the extent of LILRB2-mediated modulation of antigen-presenting cell phenotype (63). A subsequent comparison of binding strength for different MHC-I alleles to LILRB1 and LILRB2 identified distinct preferences (17). LILRB1 has a lower affinity for some HLA-A alleles; those with Ala^193^ and Val^194^ have shown lower binding ability. Ser^207^ and Gln^253^ alleles also show weaker binding to LILRB1 and are in linkage disequilibrium with Ala^193^ and Val^194^. LILRB2 has been shown to bind most strongly to HLA-A and weakly to HLA-B alleles but with greater variability for these alleles than LILRB1. Its binding is weakest to a subset of alleles including HLA-B27 and HLA-B*5701. Some of these outliers were MHC-I alleles with known disease associations, leading to the suggestion that LILR recognition of MHC-I might influence susceptibility to, and outcome of, some viral infections or autoimmune diseases.

## LILR, MHC, and Infection

Viral infection may be regarded as the primary pathology in which MHC-I recognition is essential to achieve a successful immune response. MHC-I proteins present fragments of intracellular proteins to T cells in order to enable the lysis of infected cells, and the peptide binding specificity of particular MHC-I alleles may thus influence the course of disease. There is evidence to suggest that LILR expression is induced in response to infection (64) and can be regarded as an indicator of an effective adaptive immune response (65). Studies are now beginning to highlight the relevance of LILR in particular infections and the influence of MHC-I recognition in the process.

Distinct LILR expression profiles were found to be associated with dendritic cell dysfunction during acute HIV-1 infection (66) and with “elite” control of infection (39). As there are well-characterized associations for different MHC-I alleles with either HIV viral control or progression to AIDS (67) and given that LILR have been implicated in its disease pathology, this viral infection represented a suitable model for testing the hypothesis that LILR may mediate MHC-I disease associations. Support for this theory was provided by studies, which demonstrated that MHC-I alleles and complexes associated with disease progression were preferential ligands for the inhibitory receptor LILRB2, whereas those associated with delayed onset of AIDS showed weaker binding to the receptor (17, 63, 68, 69). It could therefore be hypothesized that weaker affinity for LILRB2 would result in a lack of inhibition of dendritic cell functions, resulting in a more effective anti-HIV immune response. One study sought to examine the MHC-I haplotype of HIV-1 patient cohorts in combination with the strength of their LILR binding in order to assess whether LILR recognition might influence the course of disease. An association with LILRB2, but not LILRB1, binding strength was observed, indicating that the strength of MHC-I recognition correlates with control of viral load (18). This study provided the first strong evidence that, despite the broad specificity of LILR, the strength of their binding preference for different MHC-I alleles could represent a novel mechanism for an MHC-I association during infection.

Binding of MHC-I by “Activating” members of the LILR family may also be relevant in HIV-1 infection. LILRA1 and LILRA3 preferentially bind HLA-C open confomers (17), and HLA-C variants have been associated with different outcomes of HIV infection. One particular polymorphism, *−35C/T*, lies 35 kb upstream of the HLA-C locus. The −35C allele corresponds with increased HLA-C expression, which in turn is associated with delayed onset of AIDS (70). HLA-C proteins are more stable in open conformer form than their HLA-A and -B counterparts and are upregulated following immune cell activation. It is, therefore, possible that LILRA1 or LILRA3 recognition of HLA-C might provide a further mechanism for MHC-I disease associations during HIV infection.

Leukocyte Ig-like receptor binding preferences for MHC-I alleles may influence the outcome of other viral infections. Expression of HLA-B27 is associated with spontaneous clearance of hepatitis C virus infection (71), and by analogy with HIV-1, it could be hypothesized that the low binding preference of LILRB2 for this allele might influence disease outcome. Another viral infection where LILR may be responsible for MHC-I-associated protective effects is dengue. Large case-control studies have identified MHC-I alleles with protective effects in dengue infection (72). Antibody opsonized dengue has recently been shown to co-ligate the inhibitory receptor LILRB1 when engaged by FcγR, leading to inhibition of FcγR signaling (73) and indicating that LILRB1 may play a role in antibody-dependent dengue. Infection with DENV is highly inflammatory and results in a large influx of activated B-cells.

## Autoimmunity

Individual LILR have been implicated in autoimmunity, and their preferences for MHC-I alleles may be relevant in these conditions. Of the receptors known to recognize MHC-I, LILRA3 has been found to be associated with a number of inflammatory conditions. Expressed only in a soluble form, LILRA3 possesses no known signaling capacity of its own but can bind ligands of cell-associated LILR. Some individuals do not express LILRA3 due to a large 6.7 kbp sequence deletion. The prevalence of this deletion polymorphism is population-dependent and ranges from 6 to 84% (74, 75), with a particularly high relevance in the Japanese population, where a number of non-functional spliced isoforms have also been identified (76). The deletion has been associated with increased susceptibility and early onset of multiple sclerosis (MS) symptoms in a number of studies (77, 78), although conflicting data have been observed in other populations (74).

LILRA3 deficiency may also be a risk factor for Sjögrens syndrome (SS), with increased prevalence of null allele homozygous individuals (79) in certain populations, while the functional allele is a suggested risk factor in others (75). More recent studies have linked LILRA3 to rheumatoid arthritis (RA). In contrast to MS, increased serum level of functional LILRA3 is a proposed genetic risk factor for RA, with serum levels correlating directly with disease severity (80). Of further note is the prominent expression of LILRA2, A5, B2, and B3 in synovial tissues of RA patients (81), and the reduction of LILRA2, LILRB2, and LILRB3 in patients responsive to disease-modifying antirheumatic drugs (DMARDs) (82). Functional LILRA3 has also been suggested as a risk factor for systemic lupus erythematosus (SLE) following a genotyping study in Han Chinese populations, which also found higher levels of LILRA3 mRNA in SLE patients (75).

## Other Ligands and Functions of LILR

Direct recognition of dengue virus by LILRB1 highlights the relevance of future studies to characterize the full range of ligands for these receptors and compare their relative binding strengths. As described above, LILRB2 is known to be the most promiscuous receptor in the family in terms of its broad specificity for classical and non-classical MHC-I in folded and unfolded forms. LILRB2 has also been shown to bind a range of non-MHC ligands including angiopoietin-like proteins (32) and NOGO, a myelin component (30). More recently, LILRA3 has also been shown to bind NOGO (83). These findings extend the relevance of LILR beyond immune responses to situations such as neurodegeneration, neural plasticity, angiogenesis, and other, as yet, unidentified scenarios where MHC-I may compete with other ligands for receptor binding (84). In the future, comparative binding assays may indicate how MHC-I allelic preferences might influence the ability of LILR to bind alternative ligands. Such investigations could cast light on previous observations regarding the relevance of MHC-I in neural plasticity and regeneration (85, 86) and associations with non-immune conditions such as Alzheimer’s disease.

## Future Directions

**Figure 2:**
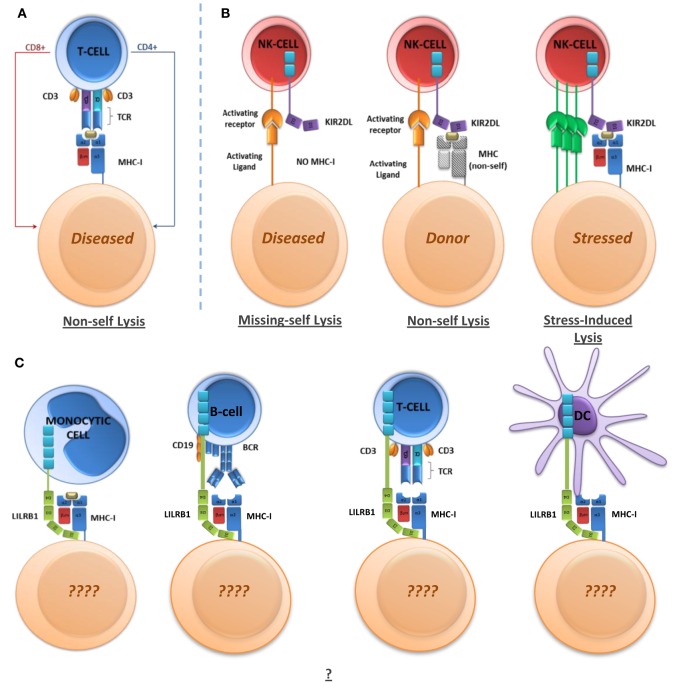
**Immunoregulatory receptor mechanisms and functions**. **(A)** T-cell-mediated non-self killing through non-self MHC-I peptide presentation. **(B)** NK-mediated non-self killing through missing-self, non-self, and stress/damage-induced lysis. **(C)** LILR-mediated regulation of immune cells. LILR may regulate cell phenotype and functions in a variety of ways, which have yet to be determined in full.

Studies on HIV-1 have provided proof of concept that LILR binding preferences for MHC-I alleles could represent a novel mechanism to explain some of the associations of MHC-I alleles with autoimmune diseases and the outcome of certain viral infections. According to this model, the influence of LILR can vary according to the strength of their binding to MHC-I alleles, representing a “degrees of self” model. MHC polymorphisms could, therefore, determine the degree of LILR signaling and consequent regulation of functions for a range of immune cell subsets as indicated in Figure 2. However, identification of the underlying mechanisms through which LILR might alter disease outcomes will require an enhanced understanding of LILR biology. It will be necessary to obtain a full characterization of the LILR expression repertoire on immune cell subsets and identify the functional effects of LILR on each cell type. For example, in the context of dengue infection, LILR expression on B cell subsets may also be relevant in viral uptake and/or generation of non-neutralizing antibodies. It will also be necessary to characterize LILR expression and function on non-immune cells. Comparative binding assays between MHC-I alleles and alternative ligands should then help to explain the wide-ranging influence of these proteins.

## Author Contributions

RA and LH were responsible for the drafting and editing of the manuscript.

## Conflict of Interest Statement

The authors declare that the research was conducted in the absence of any commercial or financial relationships that could be construed as a potential conflict of interest.

## References

[1] Robinson J Halliwell JA Hayhurst JD Flicek P Parham P Marsh SGE The IPD and IMGT/HLA database: allele variant databases. Nucleic Acids Res (2014) 43:D423–31. 10.1093/nar/gku1161 25414341 PMC4383959

[2] Brown MA Pile KD Kennedy LG Calin A Darke C Bell J HLA class I associations of ankylosing spondylitis in the white population in the United Kingdom. Ann Rheum Dis (1996) 55:268–70. 10.1136/ard.55.4.268 8733445 PMC1010149

[3] Kløverpris HN Harndahl M Leslie AJ Carlson JM Ismail N van der Stok M HIV control through a single nucleotide on the HLA-B locus. J Virol (2012) 86:11493–500. 10.1128/JVI.01020-12 22896606 PMC3486337

[4] Salloum S Oniangue-Ndza C Neumann-Haefelin C Hudson L Giuigliano S aus dem Siepen M Escape from HLA-B*08-restricted CD8 T cells by hepatitis C virus is associated with fitness costs. J Virol (2008) 82:11803–12. 10.1128/JVI.00997-08 18815309 PMC2583685

[5] Fitzmaurice K Hurst J Dring M Rauch A McLaren PJ Gunthard HF Additive effects of HLA alleles and innate immune genes determine viral outcome in HCV infection. Gut (2015) 64:813–9. 10.1136/gutjnl-2013-306287 24996883 PMC4392199

[6] Vejbaesya S Thongpradit R Kalayanarooj S Luangtrakool K Luangtrakool P Gibbons RV HLA class I supertype associations with clinical outcome of secondary dengue virus infections in ethnic thais. J Infect Dis (2015) 212:939–47. 10.1093/infdis/jiv127 25740956 PMC4548457

[7] Frater AJ Brown H Oxenius A Gunthard HF Hirschel B Robinson N Effective T-cell responses select human immunodeficiency virus mutants and slow disease progression. J Virol (2007) 81:6742–51. 10.1128/JVI.00022-07 17409157 PMC1900110

[8] Nitschke K Barriga A Schmidt J Timm J Viazov S Kuntzen T HLA-B*27 subtype specificity determines targeting and viral evolution of a hepatitis C virus-specific CD8+ T-cell epitope. J Hepatol (2014) 60:22–9. 10.1016/j.jhep.2013.08.009 23978718 PMC3867523

[9] de Castro JA. HLA-B27-bound peptide repertoires: their nature, origin and pathogenetic relevance. Adv Exp Med Biol (2009) 649:196–209. 10.1007/978-1-4419-0298-6_14 19731630

[10] Schneidewind A Brockman MA Yang R Adam RI Li B Le Gall S Escape from the dominant HLA-B27-restricted cytotoxic T-lymphocyte response in Gag is associated with a dramatic reduction in human immunodeficiency virus type 1 replication. J Virol (2007) 81:12382–93. 10.1128/JVI.01543-07 17804494 PMC2169010

[11] Peruzzi M Wagtmann N Long EO. A p70 killer cell inhibitory receptor specific for several HLA-B allotypes discriminates among peptides bound to HLA-B*2705. J Exp Med (1996) 184:1585–90. 10.1084/jem.184.4.1585 8879234 PMC2192820

[12] Wong-Baeza I Ridley A Shaw J Hatano H Rysnik O McHugh K KIR3DL2 binds to HLA-B27 dimers and free H chains more strongly than other HLA class I and promotes the expansion of T cells in ankylosing spondylitis. J Immunol (2013) 190:3216–24. 10.4049/jimmunol.1202926 23440420 PMC3736094

[13] Van Teijlingen NH Holzemer A Korner C Garcia-Beltran WF Schafer JL Fadda L Sequence variations in HIV-1 p24 Gag-derived epitopes can alter binding of KIR2DL2 to HLA-C*03:04 and modulate primary NK cell function. AIDS (2014) 28:1399–408. 10.1097/QAD.0000000000000284 24785948 PMC4453925

[14] Martin MP Carrington M. Immunogenetics of HIV disease. Immunol Rev (2013) 254:245–64. 10.1111/imr.12071 23772624 PMC3703621

[15] Borges L Hsu ML Fanger N Kubin M Cosman D. A family of human lymphoid and myeloid Ig-like receptors, some of which bind to MHC class I molecules. J Immunol (1997) 159:5192–6. 9548455

[16] Burshtyn DN Morcos C. The expanding spectrum of ligands for leukocyte Ig-like receptors. J Immunol (2016) 196:947–55. 10.4049/jimmunol.1501937 26802060

[17] Jones DC Kosmoliaptsis V Apps R Lapaque N Smith I Kono A HLA class I allelic sequence and conformation regulate leukocyte Ig-like receptor binding. J Immunol (2011) 186:2990–7. 10.4049/jimmunol.1003078 21270408

[18] Bashirova AA Martin-Gayo E Jones DC Qi Y Apps R Burke PS LILRB2 interaction with HLA class I correlates with control of HIV-1 infection. PLoS Genet (2014) 10:e1004196. 10.1371/journal.pgen.1004196 24603468 PMC3945438

[19] Barrow AD Trowsdale J. The extended human leukocyte receptor complex: diverse ways of modulating immune responses. Immunol Rev (2008) 224:98–123. 10.1111/j.1600-065X.2008.00653.x 18759923

[20] Lu HK Rentero C Raftery MJ Borges L Bryant K Tedla N Leukocyte Ig-like receptor B4 (LILRB4) is a potent inhibitor of FcγRI-mediated monocyte activation via dephosphorylation of multiple kinases. J Biol Chem (2009) 284:34839–48. 10.1074/jbc.M109.035683 19833736 PMC2787346

[21] Pilsbury LE Allen RL Vordermeier M. Modulation of toll-like receptor activity by leukocyte Ig-like receptors and their effects during bacterial infection. Mediators Inflamm (2010) 2010:536478. 10.1155/2010/536478 20634939 PMC2903975

[22] Liang S Ristich V Arase H Dausset J Carosella ED Horuzsko A Modulation of dendritic cell differentiation by HLA-G and ILT4 requires the IL-6-STAT3 signaling pathway. Proc Natl Acad Sci USA (2008) 105:8357–62. 10.1073/pnas.0803341105 18550825 PMC2448841

[23] Ketroussi F Giuliani M Bahri R Azzarone B Charpentier B Durrbach A. Lymphocyte cell-cycle inhibition by HLA-G is mediated by phosphatase SHP-2 and acts on the mTOR pathway. PLoS One (2011) 6:e22776. 10.1371/journal.pone.0022776 21887223 PMC3160837

[24] Kubagawa H Chen CC Ho LH Shimada TS Gartland L Machburn C Biochemical nature and cellular distribution of the paired immunoglobulin-like receptors, PIR-A and PIR-B. J Exp Med (1999) 189:309–18. 10.1084/jem.189.2.309 9892613 PMC2192985

[25] Liang S Baibakov B Horuzsko A. HLA-G inhibits the functions of murine dendritic cells via the PIR-B immune inhibitory receptor. Eur J Immunol (2002) 32:2418–26. 10.1002/1521-4141(200209)32:9<2418::AID-IMMU2418>3.0.CO;2-L 12207326

[26] Hogan L Bhuju S Jones DC Laing K Trowsdale J Butcher P Characterisation of bovine leukocyte Ig-like receptors. PLoS One (2012) 7:e34291. 10.1371/journal.pone.0034291 22485161 PMC3317502

[27] Dennis G Kubagawa H Cooper MD. Paired Ig-like receptor homologs in birds and mammals share a common ancestor with mammalian Fc receptors. Proc Natl Acad Sci USA (2000) 97:13245–50. 10.1073/pnas.230442897 11078516 PMC27210

[28] Stafford JL Bengten E Du Pasquier L McIntosh RD Quiniou SM Clem LW A novel family of diversified immunoregulatory receptors in teleosts is homologous to both mammalian Fc receptors and molecules encoded within the leukocyte receptor complex. Immunogenetics (2006) 58:758–73. 10.1007/s00251-006-0134-1 16845542 PMC1592254

[29] Takai T Nakamura A Endo S. Role of PIR-B in autoimmune glomerulonephritis. J Biomed Biotechnol (2011) 2011:275302. 10.1155/2011/275302 20976309 PMC2952822

[30] Kim T Vidal GS Djurisic M William CM Birnbaum ME Garcia KC Human LilrB2 is a β-amyloid receptor and its murine homolog PirB regulates synaptic plasticity in an Alzheimer’s model. Science (2013) 341:1399–404. 10.1126/science.1242077 24052308 PMC3853120

[31] Fan X Shi P dai J Lu Y Chen X Liu X Paired immunoglobulin-like receptor B regulates platelet activation. Blood (2014) 124:2421–30. 10.1182/blood-2014-03-557645 25075127 PMC4192752

[32] Zheng J Umikawa M Cui C Li J Chen X Zhang C Inhibitory receptors bind ANGPTLs and support blood stem cells and leukaemia development. Nature (2012) 485:656–60. 10.1038/nature11095 22660330 PMC3367397

[33] Tedla N Lee CW Borges L Geczy CL Arm JP Differential expression of leukocyte immunoglobulin-like receptors on cord blood-derived human mast cell progenitors and mature mast cells. J Leukoc Biol (2008) 83:334–43. 10.1189/jlb.0507314 17998301

[34] McIntire RH Sofers T Platt JS Ganacias KG Langat DK Hunt JS. Novel HLA-G-binding leukocyte immunoglobulin-like receptor (LILR) expression patterns in human placentas and umbilical cords. Placenta (2008) 29:631–8. 10.1016/j.placenta.2008.04.007 18538388 PMC2505055

[35] Moir S Ho J Malaspina A Wang W DiPoto AC O’Shea MA Evidence for HIV-associated B cell exhaustion in a dysfunctional memory B cell compartment in HIV-infected viremic individuals. J Exp Med (2008) 205:1797–805. 10.1084/jem.20072683 18625747 PMC2525604

[36] Brown D Jones DC Anderson KJ Lapaque N Buerki RA Trowsdale J The inhibitory receptor LILRB4 (ILT3) modulates antigen presenting cell phenotype and, along with LILRB2 (ILT4), is upregulated in response to Salmonella infection. BMC Immunol (2009) 10:56. 10.1186/1471-2172-10-56 19860908 PMC2773765

[37] Mori Y Tsuji S Sakamoto Y Endo S Ito Y Fujimura S Inhibitory immunoglobulin-like receptors LILRB and PIR-B negatively regulate osteoclast development. J Immunol (2008) 181:4742–51. 10.4049/jimmunol.181.7.4742 18802077

[38] Lu HK Mitchell A Endoh Y Hampartzoumian T Huynh O Borges L LILRA2 selectively modulates LPS-mediated cytokine production and inhibits phagocytosis by monocytes. PLoS One (2012) 7:e33478. 10.1371/journal.pone.0033478 22479404 PMC3316576

[39] Huang J Burke PS Cung TD Pereyra F Toth I Walker BD Leukocyte immunoglobulin-like receptors maintain unique antigen-presenting properties of circulating myeloid dendritic cells in HIV-1-infected elite controllers. J Virol (2010) 84:9463–71. 10.1128/JVI.01009-10 20631139 PMC2937602

[40] Brown D Trowsdale J Allen R. The LILR family: modulators of innate and adaptive immune pathways in health and disease. Tissue Antigens (2004) 64:215–25. 10.1111/j.0001-2815.2004.00290.x 15304001

[41] Cao W Bover L Cho M Wen X Hanabuchi S Bao M Regulation of TLR7/9 responses in plasmacytoid dendritic cells by BST2 and ILT7 receptor interaction. J Exp Med (2009) 206:1603–14. 10.1084/jem.20090547 19564354 PMC2715090

[42] Torii I Oka S Hotomi M Benjamin WH Jr Takai T Kearney JF PIR-B deficient mice are susceptible to salmonella infection. J Immunol (2008) 181:4229–39. 10.4049/jimmunol.181.6.4229 18768880 PMC2613810

[43] Bleharski JR Li H Meinken C Graeber TG Ochoa MT Yamamura M Use of genetic profiling in leprosy to discriminate clinical forms of the disease. Science (2003) 301:1527–30. 10.1126/science.1087785 12970564

[44] Young NT Waller EC Patel R Roghanian A Austyn JM Trowsdale J. The inhibitory receptor LILRB1 modulates the differentiation and regulatory potential of human dendritic cells. Blood (2008) 111:3090–6. 10.1182/blood-2007-05-089771 18094328

[45] Chang CC Ciubotariu R Manavalan JS Yuan J Colovai AI Piazza F Tolerization of dendritic cells by TS cells: the crucial role of inhibitory receptors ILT3 and ILT4. Nat Immunol (2002) 3:237–43. 10.1038/ni760 11875462

[46] Huang J Burke P Yang Y Seiss K Beamon J Cung T Soluble HLA-G inhibits myeloid dendritic cell function in hiv-1 infection by interacting with leukocyte immunoglobulin-like receptor B2. J Virol (2010) 84:10784–91. 10.1128/JVI.01292-10 20702625 PMC2950595

[47] Brenk M Scheler M Kocj S Neumann J Takikawa O Hacker G Tryptophan deprivation induces inhibitory receptors ILT3 and ILT4 on dendritic cells favoring the induction of human CD4+CD25+ Foxp3+ T regulatory cells. J Immunol (2009) 183:145–54. 10.4049/jimmunol.0803277 19535644

[48] Gregori S Tomasoni D Pacciani V Scripoli M Battaglia M Magnani CF Differentiation of type 1 T regulatory cells (Tr1) by tolerogenic DC-10 requires the IL-10–dependent ILT4/HLA-G pathway. Blood (2010) 116:935–44. 10.1182/blood-2009-07-234872 20448110

[49] Stallone G Pontrelli P Infante B Gigante M Netti GS Ranieri E Rapamycin induces ILT3(high)ILT4(high) dendritic cells promoting a new immunoregulatory pathway. Kidney Int (2014) 85:888–97. 10.1038/ki.2013.337 24107844

[50] Banchereau J Zurawski S Thompson-Snipes L Blanck JP Clayton S Munk A Immunoglobulin-like transcript receptors on human dermal CD14+ dendritic cells act as a CD8-antagonist to control cytotoxic T cell priming. Proc Natl Acad Sci USA (2012) 109:18885–90. 10.1073/pnas.1205785109 23112154 PMC3503187

[51] Merlo A Tenca C Fais F Battini L Ciccone E Grossi CE Inhibitory receptors CD85j, LAIR-1, and CD152 down-regulate immunoglobulin and cytokine production by human B lymphocytes. Clin Vaccine Immunol (2005) 12:705–12. 10.1128/CDLI.12.6.705-712.2005 15939744 PMC1151979

[52] Dietrich J Cella M Colonna M. Ig-like transcript 2 (ILT2)/leukocyte Ig-like receptor 1 (LIR1) inhibits TCR signaling and actin cytoskeleton reorganization. J Immunol (2001) 166:2514–21. 10.4049/jimmunol.166.4.2514 11160312

[53] Colonna M Navarro F Bellon T Llano M Garcia P Samaridis J A common inhibitory receptor for major histocompatibility complex class I molecules on human lymphoid and myelomonocytic cells. J Exp Med (1997) 186:1809–18. 10.1084/jem.186.11.1809 9382880 PMC2199153

[54] Mitchell A Rentero C Endoh Y Hsu K Gaus K Geczy C LILRA5 is expressed by synovial tissue macrophages in rheumatoid arthritis, selectively induces pro-inflammatory cytokines and IL-10 and is regulated by TNF-α, IL-10 and IFN-γ. Eur J Immunol (2008) 38:3459–73. 10.1002/eji.200838415 19009525

[55] Sloane DE Tedla N Awoniyi M Macglashan DW Jr Borges L Austen KF Leukocyte immunoglobulin-like receptors: novel innate receptors for human basophil activation and inhibition. Blood (2004) 104:2832–9. 10.1182/blood-2004-01-0268 15242876

[56] Cosman D Fanger N Borges L Kubin M Chin W Peterson L A novel immunoglobulin superfamily receptor for cellular and viral MHC class I molecules. Immunity (1997) 7:273–82. 10.1016/S1074-7613(00)80529-4 9285411

[57] Willcox BE Thomas LM Bjorkman PJ. Crystal structure of HLA-A2 bound to LIR-1, a host and viral major histocompatibility complex receptor. Nat Immunol (2003) 4:913–9. 10.1038/ni961 12897781

[58] Ottonello L Ghio M Contini P Bertolotto M Bianchi G Montecucco F Nonleukoreduced red blood cell transfusion induces a sustained inhibition of neutrophil chemotaxis by stimulating in vivo production of transforming growth factor-β1 by neutrophils: role of the immunoglobulin-like transcript 1, sFasL, and sHLA-I. Transfusion (2007) 47:1395–404. 10.1111/j.1537-2995.2007.01268.x 17655583

[59] Shiroishi M Kuroki K Rasubala L Tsumoto K Kumagai I Kurimoto E Structural basis for recognition of the nonclassical MHC molecule HLA-G by the leukocyte Ig-like receptor B2 (LILRB2/LIR2/ILT4/CD85d). Proc Natl Acad Sci USA (2006) 103:16412–7. 10.1073/pnas.0605228103 17056715 PMC1637596

[60] Masuda A Nakamura A Maeda T Sakamoto Y Takai T. Cis binding between inhibitory receptors and MHC class I can regulate mast cell activation. J Exp Med (2007) 204:907–20. 10.1084/jem.20060631 17420263 PMC2118540

[61] Li NL Fu L Uchtenhagen H Achour A Burshtyn DN. Cis association of leukocyte Ig-like receptor 1 with MHC class I modulates accessibility to antibodies and HCMV UL18. Eur J Immunol (2013) 43:1042–52. 10.1002/eji.201242607 23348966

[62] Finton KA Strong RK. Structural insights into activation of antiviral NK cell responses. Immunol Rev (2012) 250:239–57. 10.1111/j.1600-065X.2012.01168.x 23046134 PMC3471384

[63] Lichterfeld M Kavanagh DG Williams KL Moza B Mui SK Miura T A viral CTL escape mutation leading to immunoglobulin-like transcript 4–mediated functional inhibition of myelomonocytic cells. J Exp Med (2007) 204:2813–24. 10.1084/jem.20061865 18025130 PMC2118510

[64] Smith CL Dickinson P Forster T Craigon M Ross A Khondoker MR Identification of a human neonatal immune-metabolic network associated with bacterial infection. Nat Commun (2014) 14:4649. 10.1038/ncomms5649 25120092 PMC4143936

[65] Nakaya HI Wrammert J Lee EK Racioppi L Markie-Kunze S Haining WN Systems biology of seasonal influenza vaccination in humans. Nat Immunol (2011) 12:786–95. 10.1038/ni.2067 21743478 PMC3140559

[66] Huang J Yang Y Al-Mozaini M Burke PS Beamon J Carrington MF Dendritic cell dysfunction during primary HIV-1 infection. J Infect Dis (2011) 204:1557–62. 10.1093/infdis/jir616 21969335 PMC3192192

[67] The International HIV Controllers Study Pereyra F Jia X Mclaren PJ Tlenti A De Bakker PI The major genetic determinants of HIV-1 control affect HLA class I peptide presentation. Science (2010) 330:1551–7. 10.1126/science.1195271 21051598 PMC3235490

[68] Yang Y Huang J Toth I Lichterfeld M Yu XG. Mutational escape in HIV-1 CTL epitopes leads to increased binding to inhibitory myelomonocytic MHC class I receptors. PLoS One (2010) 5:e15084. 10.1371/journal.pone.0015084 21170342 PMC2999561

[69] Huang J Goedert JJ Sundberg EJ Cung TD Burke PS Martin MP HLA-B*35-Px–mediated acceleration of HIV-1 infection by increased inhibitory immunoregulatory impulses. J Exp Med (2009) 206:2959–66. 10.1084/jem.20091386 20008523 PMC2806456

[70] Thomas R Apps R Qi Y Gao X Male V O’hUigin C HLA-C cell surface expression and control of HIV/AIDS correlate with a variant upstream of HLA-C. Nat Genet (2009) 41:1290–4. 10.1038/ng.486 19935663 PMC2887091

[71] Bengsch B Thimme R Blum HE. Role of host genetic factors in the outcome of hepatitis C virus infection. Viruses (2009) 1:104–25. 10.3390/v1020104 21994541 PMC3185494

[72] Stephens HA. HLA and other gene associations with dengue disease severity. Curr Top Microbiol Immunol (2010) 338:99–114. 10.1007/978-3-642-02215-9_8 19802581

[73] Chan KR Ong EZ Tan HC Zhang SL Zhang Q Tang KF Leukocyte immunoglobulin-like receptor B1 is critical for antibody-dependent dengue. Proc Natl Acad Sci USA (2014) 111:2722–7. 10.1073/pnas.1317454111 24550301 PMC3932915

[74] Wiśniewski A Wagner M Nowak I Bilinska M Pokryszko-Dragan A Jasek M 6.7-kbp deletion in LILRA3 (ILT6) gene is associated with later onset of the multiple sclerosis in a Polish population. Hum Immunol (2013) 74:353–7. 10.1016/j.humimm.2012.12.006 23238213

[75] Du Y Su Y He J Yang Y Shi Y Cui Y Impact of the leucocyte immunoglobulin-like receptor A3 (LILRA3) on susceptibility and subphenotypes of systemic lupus erythematosus and Sjögren’s syndrome. Ann Rheum Dis (2015) 74:2070–5. 10.1136/annrheumdis-2013-204441 24906639

[76] Hirayasu K Ohashi J Kashiwase K Takanashi M Satake M Tokunaga K Long-term persistence of both functional and non-functional alleles at the leukocyte immunoglobulin-like receptor A3 (LILRA3) locus suggests balancing selection. Hum Genet (2006) 119:436–43. 10.1007/s00439-006-0152-y 16501917

[77] Ordonez D Sanchez AJ Martinez-Rodriquez JE Cisneros E Ramil E Romo N Multiple sclerosis associates with LILRA3 deletion in Spanish patients. Genes Immun (2009) 10:579–85. 10.1038/gene.2009.34 19421224

[78] Koch S Goedde R Migmatova V Epplen JT Muller N de Seze J Association of multiple sclerosis with ILT6 deficiency. Genes Immun (2005) 6:445–7. 10.1038/sj.gene.6364187 15815690

[79] Kabalak G Dobberstein SB Matthias T Reuter S The YH Dorner T Association of immunoglobulin-like transcript 6 deficiency with Sjögren’s syndrome. Arthritis Rheum (2009) 60:2923–5. 10.1002/art.24804 19790059

[80] An H Chandra V Piraino B Borges L Geczy C McNeil HP Soluble LILRA3, a potential natural antiinflammatory protein, is increased in patients with rheumatoid arthritis and is tightly regulated by interleukin 10, tumor necrosis factor-α, and interferon-γ. J Rheumatol (2010) 37:1596–606. 10.3899/jrheum.091119 20595277

[81] Tedla N An H Borges L Vollmer-Conna U Bryant K Geczy C Expression of activating and inhibitory leukocyte immunoglobulin-like receptors in rheumatoid synovium: correlations to disease activity. Tissue Antigens (2011) 77:305–16. 10.1111/j.1399-0039.2011.01633.x 21388353

[82] Huynh OA Hampartzoumian T Arm JP Hunt J Borges L Ahern M Down-regulation of leucocyte immunoglobulin-like receptor expression in the synovium of rheumatoid arthritis patients after treatment with disease-modifying anti-rheumatic drugs. Rheumatology (2007) 46:742–51. 10.1093/rheumatology/kel405 17202177

[83] An H Brettle M Lee T Heng B Lim CK Guillemin GJ Soluble LILRA3 promotes neurite outgrowth and synapses formation through high affinity interaction with Nogo 66. J Cell Sci (2016) 129:1198–209. 10.1242/jcs.182006 26826187

[84] Matsushita H Endo S Kobayashi E Sakamoto Y Kobayashi K Kitaguchi K Differential but competitive binding of nogo protein and class I major histocompatibility complex (MHCI) to the PIR-B ectodomain provides an inhibition of cells. J Biol Chem (2011) 286:25739–47. 10.1074/jbc.M110.157859 21636572 PMC3138294

[85] Cebrián C Loike JD Sulzer D Neuronal MHC-I expression and its implications in synaptic function, axonal regeneration and Parkinson’s and other brain diseases. Front Neuroanat (2014) 8:114 10.3389/fnana.2014.00114 25352786 PMC4195363

[86] Debnath M Cannon DM Venkatasubramanian G. Variation in the major histocompatibility complex [MHC] gene family in schizophrenia: associations and functional implications. Prog Neuropsychopharmacol Biol Psychiatry (2013) 42:49–62. 10.1016/j.pnpbp.2012.07.009 22813842

